# Supplementary material for: A multilayered genetic structure analysis between inflammatory bowel disease and bone density/osteoporosis
Source: PLoS One. 2025 Nov 20;20(11):e0336775. doi: 10.1371/journal.pone.0336775 (PMC12633866; doi:10.1371/journal.pone.0336775)
Supplement: S1 File — (DOCX) [file pone.0336775.s002.docx]

**STROBE-MR checklist of recommended items to address in reports of Mendelian randomization studies**^1^ ^2^

| **Item No.** | **Section** | **Checklist item** | **Page No.** | **Relevant text from manuscript** |
| --- | --- | --- | --- | --- |
| 1 | **TITLE and ABSTRACT** | Indicate Mendelian randomization (MR) as the study’s design in the title and/or the abstract if that is a main purpose of the study | **1** | Mendelian randomization (MR) analysis was performed to infer potential causal relationships. |
|  | **INTRODUCTION** |  |  |  |
| 2 | **Background** | Explain the scientific background and rationale for the reported study. What is the exposure? Is a potential causal relationship between exposure and outcome plausible? Justify why MR is a helpful method to address the study question | **2** | IBD patients may endure lifelong challenges from ongoing inflammation and related complications, one of which is reduced bone mineral density (BMD), thereby heightening the risk of osteoporosis [5–7]. Recent Mendelian randomization studies have revealed significant causal relationships between IBD and both osteoporosis and BMD |
| 3 | **Objectives** | State specific objectives clearly, including pre-specified causal hypotheses (if any). State that MR is a method that, under specific assumptions, intends to estimate causal effects | **4** | Mendelian randomization (MR) analysis was performed to infer potential causal relationships |
|  | **METHODS** |  |  |  |
| 4 | **Study design and data sources** | Present key elements of the study design early in the article. Consider including a table listing sources of data for all phases of the study. For each data source contributing to the analysis, describe the following: |  |  |
|  | a) | Setting: Describe the study design and the underlying population, if possible. Describe the setting, locations, and relevant dates, including periods of recruitment, exposure, follow-up, and data collection, when available. | **NA** | NA |
|  | b) | Participants: Give the eligibility criteria, and the sources and methods of selection of participants. Report the sample size, and whether any power or sample size calculations were carried out prior to the main analysis | **4** | The GWAS results for IBD (Ncase=25,042, Ncontrol=34,915, Ntotal=59,957) and its subtypes (CD (Ncase=12,194, Ncontrol=28,072, Ntotal=40,266) and UC (Ncase=12,366, Ncontrol=33,609, Ntotal=45,975)) were sourced from the study by de Lange KM et al. [22]. Data pertaining to BMD was obtained from a meta-analysis examining total BMD (Ntotal=31,4921) and age-related effects [23]. The osteoporosis data (Ncase=8,017, Ncontrol=391,037, Ntotal=399,054) were extracted from the FinnGen database (https://r10.finngen.fi/) |
|  | c) | Describe measurement, quality control and selection of genetic variants | **NA** | NA |
|  | d) | For each exposure, outcome, and other relevant variables, describe methods of assessment and diagnostic criteria for diseases | **NA** | NA |
|  | e) | Provide details of ethics committee approval and participant informed consent, if relevant | **Not involved** | Not involved |
| 5 | **Assumptions** | Explicitly state the three core IV assumptions for the main analysis (relevance, independence and exclusion restriction) as well assumptions for any additional or sensitivity analysis | **6** | The analysis strictly adhered to the three core assumptions of MR |
| 6 | **Statistical methods: main analysis** | Describe statistical methods and statistics used |  |  |
|  | a) | Describe how quantitative variables were handled in the analyses (i.e., scale, units, model) | **6-7** | In the genome-wide analysis, we performed variant selection and applied linkage disequilibrium (LD) clumping, with the following thresholds: P-value < 5 × 10⁻⁸, LD r² = 0.001, and genomic distance of 10,000 kbp.  To ensure the robustness of the results, we conducted sensitivity analyses, including tests for horizontal pleiotropy [29,30], heterogeneity analysis [31], and leave-one-out validation [32]. |
|  | b) | Describe how genetic variants were handled in the analyses and, if applicable, how their weights were selected | **6-7** | In the genome-wide analysis, we performed variant selection and applied linkage disequilibrium (LD) clumping, with the following thresholds: P-value < 5 × 10⁻⁸, LD r² = 0.001, and genomic distance of 10,000 kbp.  To ensure the robustness of the results, we conducted sensitivity analyses, including tests for horizontal pleiotropy [29,30], heterogeneity analysis [31], and leave-one-out validation [32]. All analyses were performed in the R environment, primarily using the TwoSampleMR package (https://mrcieu.github.io/TwoSampleMR/) and the MR-PRESSO package (https://github.com/rondolab/MR-PRESSO) for data analysis and causal inference. |
|  | c) | Describe the MR estimator (e.g. two-stage least squares, Wald ratio) and related statistics. Detail the included covariates and, in case of two-sample MR, whether the same covariate set was used for adjustment in the two samples | **6-7** | In the genome-wide analysis, we performed variant selection and applied linkage disequilibrium (LD) clumping, with the following thresholds: P-value < 5 × 10⁻⁸, LD r² = 0.001, and genomic distance of 10,000 kbp.  To ensure the robustness of the results, we conducted sensitivity analyses, including tests for horizontal pleiotropy [29,30], heterogeneity analysis [31], and leave-one-out validation [32]. All analyses were performed in the R environment, primarily using the TwoSampleMR package (https://mrcieu.github.io/TwoSampleMR/) and the MR-PRESSO package (https://github.com/rondolab/MR-PRESSO) for data analysis and causal inference. |
|  | d) | Explain how missing data were addressed | **Not involved** |  |
|  | e) | If applicable, indicate how multiple testing was addressed | **Not involved** |  |
| 7 | **Assessment of assumptions** | Describe any methods or prior knowledge used to assess the assumptions or justify their validity | **6** | The analysis strictly adhered to the three core assumptions of MR |
| 8 | **Sensitivity analyses and additional analyses** | Describe any sensitivity analyses or additional analyses performed (e.g. comparison of effect estimates from different approaches, independent replication, bias analytic techniques, validation of instruments, simulations) | **7** | To ensure the robustness of the results, we conducted sensitivity analyses, including tests for horizontal pleiotropy [29,30], heterogeneity analysis [31], and leave-one-out validation [32]. All analyses were performed in the R environment, primarily using the TwoSampleMR package (https://mrcieu.github.io/TwoSampleMR/) and the MR-PRESSO package (https://github.com/rondolab/MR-PRESSO) for data analysis and causal inference. |
| 9 | **Software and pre-registration** |  |  |  |
|  | a) | Name statistical software and package(s), including version and settings used | **16** | 1. All the GWAS data and statistical software used in this study were publicly available (which can be accessed through the following URLs), and all the generated results in this study were provided in the main text and supplemental data. |
|  | b) | State whether the study protocol and details were pre-registered (as well as when and where) | **Not involved** | Not involved |
|  | **RESULTS** |  |  |  |
| 10 | **Descriptive data** |  |  |  |
|  | a) | Report the numbers of individuals at each stage of included studies and reasons for exclusion. Consider use of a flow diagram | **4** | To ensure the reliability and validity of the genetic analysis results, we implemented a strict quality control process. Variants included in the final analysis had to meet the following criteria: First, variant screening and calibration were based on the 1000 Genomes Project Phase 3 data as the reference panel. Second, only biallelic variants were retained, with the minor allele frequency (MAF) for European population data set above 0.01 to ensure statistical power. Finally, all variants were accurately annotated to their genomic positions based on the human reference genome (hg19/GRCh37). Variants lacking rsID identifiers or with conflicting rsID annotations were excluded to ensure data accuracy. |
|  | b) | Report summary statistics for phenotypic exposure(s), outcome(s), and other relevant variables (e.g. means, SDs, proportions) | **4** | The GWAS results for IBD (Ncase=25,042, Ncontrol=34,915, Ntotal=59,957) and its subtypes (CD (Ncase=12,194, Ncontrol=28,072, Ntotal=40,266) and UC (Ncase=12,366, Ncontrol=33,609, Ntotal=45,975)) were sourced from the study by de Lange KM et al. [22]. Data pertaining to BMD was obtained from a meta-analysis examining total BMD (Ntotal=31,4921) and age-related effects [23]. The osteoporosis data (Ncase=8,017, Ncontrol=391,037, Ntotal=399,054) were extracted from the FinnGen database (https://r10.finngen.fi/) [24]. |
|  | c) | If the data sources include meta-analyses of previous studies, provide the assessments of heterogeneity across these studies | **Not involved** | Not involved |
|  | d) | For two-sample MR:  i.  Provide justification of the similarity of the genetic variant-exposure associations between the exposure and outcome samples  ii.  Provide information on the number of individuals who overlap between the exposure and outcome studies | **Not involved** | **Not involved** |
| 11 | **Main results** |  |  |  |
|  | a) | Report the associations between genetic variant and exposure, and between genetic variant and outcome, preferably on an interpretable scale | **10** | This study employed bidirectional MR analysis to investigate the causal relationships between IBD, BMD, and osteoporosis. When IBD and its subtypes were considered as exposure variables and BMD as the outcome, IBD and UC were found to be negatively associated with BMD, a result consistent with previous MR studies [10]. When osteoporosis was treated as the outcome, a positive causal association was observed between IBD and CD [8–10]. No significant causal effects were detected in the reverse analysis. |
|  | b) | Report MR estimates of the relationship between exposure and outcome, and the measures of uncertainty from the MR analysis, on an interpretable scale, such as odds ratio or relative risk per SD difference | Supplementary Table 9 | Supplementary Table 9 |
|  | c) | If relevant, consider translating estimates of relative risk into absolute risk for a meaningful time period | **NA** | NA |
|  | d) | Consider plots to visualize results (e.g. forest plot, scatterplot of associations between genetic variants and outcome versus between genetic variants and exposure) | **NA** | NA |
| 12 | **Assessment of assumptions** |  |  |  |
|  | a) | Report the assessment of the validity of the assumptions | **10-11** | Throughout all MR analyses, no evidence of horizontal pleiotropy was found, further confirming the validity and reliability of the selected instrumental variables. Additionally, all F-statistics exceeded the conventional threshold of 10, indicating that the instrumental variables were sufficiently strong and minimizing potential bias from weak instruments, thereby reinforcing the robustness of the causal estimates (Supplementary Table 7). The leave-one-out sensitivity analysis demonstrated a consistent distribution of SNP effects, with no outlier variants detected. |
|  | b) | Report any additional statistics (e.g., assessments of heterogeneity across genetic variants, such as *I^2^*, Q statistic or E-value) | **10-11** | Throughout all MR analyses, no evidence of horizontal pleiotropy was found, further confirming the validity and reliability of the selected instrumental variables. Additionally, all F-statistics exceeded the conventional threshold of 10, indicating that the instrumental variables were sufficiently strong and minimizing potential bias from weak instruments, thereby reinforcing the robustness of the causal estimates (Supplementary Table 7). |
| 13 | **Sensitivity analyses and additional analyses** |  |  |  |
|  | a) | Report any sensitivity analyses to assess the robustness of the main results to violations of the assumptions | **11** | The leave-one-out sensitivity analysis demonstrated a consistent distribution of SNP effects, with no outlier variants detected. |
|  | b) | Report results from other sensitivity analyses or additional analyses | **10-11** | Throughout all MR analyses, no evidence of horizontal pleiotropy was found, further confirming the validity and reliability of the selected instrumental variables. Additionally, all F-statistics exceeded the conventional threshold of 10, indicating that the instrumental variables were sufficiently strong and minimizing potential bias from weak instruments, thereby reinforcing the robustness of the causal estimates (Supplementary Table 7). |
|  | c) | Report any assessment of direction of causal relationship (e.g., bidirectional MR) | **NA** | NA |
|  | d) | When relevant, report and compare with estimates from non-MR analyses | **NA** | NA |
|  | e) | Consider additional plots to visualize results (e.g., leave-one-out analyses) | **11** | The leave-one-out sensitivity analysis demonstrated a consistent distribution of SNP effects, with no outlier variants detected. |
|  | **DISCUSSION** |  |  |  |
| 14 | **Key results** | Summarize key results with reference to study objectives | **10** | IBD and UC were found to be negatively associated with BMD, a result consistent with previous MR studies [10]. When osteoporosis was treated as the outcome, a positive causal association was observed between IBD and CD [8–10]. No significant causal effects were detected in the reverse analysis. |
| 15 | **Limitations** | Discuss limitations of the study, taking into account the validity of the IV assumptions, other sources of potential bias, and imprecision. Discuss both direction and magnitude of any potential bias and any efforts to address them | **15-16** | This study systematically examined the link between IBD and BMD/osteoporosis through the lenses of Rg and shared loci. Various methodologies were utilized to uncover genetic associations at both the genome-wide and SNP levels, thereby broadening the existing knowledge. Nevertheless, certain limitations warrant attention: the influence of LD could not be entirely excluded, potential sample overlap may exist, and the data were exclusively sourced from European populations, restricting cross-ethnic generalizability. The GWAS datasets used in this study were not stratified by sex, age, or disease stage, limiting our ability to explore sex-, age-, and stage-specific genetic susceptibility to IBD, BMD, and osteoporosis comorbidity. Future analyses will address this limitation if such data become available. |
| 16 | **Interpretation** |  |  |  |
|  | a) | Meaning: Give a cautious overall interpretation of results in the context of their limitations and in comparison with other studies | **15** | This study systematically examined the link between IBD and BMD/osteoporosis through the lenses of Rg and shared loci. Various methodologies were utilized to uncover genetic associations at both the genome-wide and SNP levels, thereby broadening the existing knowledge. Nevertheless, certain limitations warrant attention: the influence of LD could not be entirely excluded, potential sample overlap may exist, and the data were exclusively sourced from European populations, restricting cross-ethnic generalizability. |
|  | b) | Mechanism: Discuss underlying biological mechanisms that could drive a potential causal relationship between the investigated exposure and the outcome, and whether the gene-environment equivalence assumption is reasonable. Use causal language carefully, clarifying that IV estimates may provide causal effects only under certain assumptions | **14-15** | The Wnt signaling pathway, which was identified through enrichment analysis, warrants further investigation. As a highly conserved mechanism of signal transduction, this pathway is integral to the regulation of bone metabolism and the maintenance of intestinal homeostasis. It has been demonstrated that the canonical Wnt/β-catenin signaling pathway directly influences BMD by modulating osteoblast differentiation and proliferation |
|  | c) | Clinical relevance: Discuss whether the results have clinical or public policy relevance, and to what extent they inform effect sizes of possible interventions | **12** | For example, a survey conducted on Polish IBD patients reported that osteoporosis prevalence was 48.6% in CD patients and 33.3% in UC patients. Furthermore, IBD patients diagnosed with osteoporosis exhibited markedly lower levels of physical activity (P = 0.0335) [37]. A separate multinational prospective investigation documented that 12%-22% of IBD patients had asymptomatic vertebral fractures, with prevalence rates of 19.6% (44/224) in Canada, 21.8% (34/156) in Germany, and 12.2% (22/179) in Israel [38,39].Research by D. Leslie et al. also corroborated these findings, demonstrating that IBD patients showed reduced BMD and a markedly elevated risk of osteoporosis [40]. Additionally, analyses conducted by C. Noble et al. using both univariate and multivariate approaches indicated that a low body mass index (<18.5) was strongly associated with osteoporosis (P = 0.021, OR: 5.83, CI: 1.31-25.94) [41]. A study from Sri Lanka involving 444 participants (case-control ratio of 1:3) further revealed that DXA bone density scans indicated a markedly higher overall incidence of osteoporosis in IBD patients compared to controls (13.5% vs. 4.5%, P = 0.001) [42]. |
| 17 | **Generalizability** | Discuss the generalizability of the study results (a) to other populations, (b) across other exposure periods/timings, and (c) across other levels of exposure | **15** | This study systematically examined the link between IBD and BMD/osteoporosis through the lenses of Rg and shared loci. Various methodologies were utilized to uncover genetic associations at both the genome-wide and SNP levels, thereby broadening the existing knowledge. Nevertheless, certain limitations warrant attention: the influence of LD could not be entirely excluded, potential sample overlap may exist, and the data were exclusively sourced from European populations, restricting cross-ethnic generalizability. |
|  | **OTHER INFORMATION** |  |  |  |
| 18 | **Funding** | Describe sources of funding and the role of funders in the present study and, if applicable, sources of funding for the databases and original study or studies on which the present study is based | **17** | This study was supported by the Jiangxi Provincial Natural Science Foundation project (20232BAB206154) and the University-Level Research Project of Zhejiang Chinese Medical University (2022FSYYZQ21). The funders had no role in the study design, data collection and analysis, decision to publish, or preparation of the manuscript. |
| 19 | **Data and data sharing** | Provide the data used to perform all analyses or report where and how the data can be accessed, and reference these sources in the article. Provide the statistical code needed to reproduce the results in the article, or report whether the code is publicly accessible and if so, where |  | All GWAS data and statistical software utilized in this study were publicly available (accessible via the following URLs), and all results generated in this study have been provided in the main text and supplementary data.  IEU database: https://gwas.mrcieu.ac.uk/  GNOVA : https://github.com/xtonyjiang/GNOVA  HDL: https://github.com/zhenin/HDL  LAVA: https://github.com/josefin-werme/LAVA  TwoSampleMR: https://mrcieu.github.io/TwoSampleMR/  MR-PRESSO: https://github.com/rondolab/MR-PRESSO  ConjFDR: https://github.com/precimed/pleiofdr  FUMA: https://fuma.ctglab.nl  FinnGen: https://r12.finngen.fi/  Sangerbox: http://vip.sangerbox.com/ |
| 20 | **Conflicts of Interest** | All authors should declare all potential conflicts of interest |  | The authors declare that the research was conducted in the absence of any commercial or financial relationships that could be construe as a potential conflict of interest. |

This checklist is copyrighted by the Equator Network under the Creative Commons Attribution 3.0 Unported (CC BY 3.0) license.

1. Skrivankova VW, Richmond RC, Woolf BAR, Yarmolinsky J, Davies NM, Swanson SA, et al. Strengthening the Reporting of Observational Studies in Epidemiology using Mendelian Randomization (STROBE-MR) Statement. JAMA. 2021;under review.

2. Skrivankova VW, Richmond RC, Woolf BAR, Davies NM, Swanson SA, VanderWeele TJ, et al. Strengthening the Reporting of Observational Studies in Epidemiology using Mendelian Randomisation (STROBE-MR): Explanation and Elaboration. BMJ. 2021;375:n2233.
