# Supplementary material for: A multilayered genetic structure analysis between inflammatory bowel disease and bone density/osteoporosis
Source: PLoS One. 2025 Nov 20;20(11):e0336775. doi: 10.1371/journal.pone.0336775 (PMC12633866; doi:10.1371/journal.pone.0336775)
Supplement: Attachment — Submitted filename: To the editor.pdf [file pone.0336775.s003.pdf]

University of Baghdad/
College of Science for Women
Biology department
Baghdad
18 /7 /2025

Dear Editors in PLOS ONE

I hope you are fine.

Thank you to give me the opportunity to review the manuscript, PONE-D-25-31485, entitled (A Multilayered Genetic Structure Analysis between Inflammatory Bowel Disease and Bone Density/Osteoporosis).

The study focuses on the wide genomic data analysis of the association between inflammatory bowel disease (IBD) and reduced bone mineral density (BMD) or osteoporosis.

The study analyzes a large cohort of the UK biobank patients with Inflammatory bowel disease.

In the methods:

-The total number of participants in the study did not specify the number of males and females or the participants age distribution to identify the more affected sex and age.

- As the IBD (Crohn's disease (CD) and ulcerative colitis (UC)) is mentioned in lines 32 and 33, it is expected the immune response genes are involved, but genes that govern the immune response, like HLA genes, or genes related to the absorption and metabolism of calcium, potassium, phosphorus, etc., and genes related to vitamin D, such as VDR, etc., are not analyzed in the study, which may have an important impact on bone density.

-It will be a significant addition if further genes are included and their role in bone density is elucidated in a large number of populations and categorized according to their sex and age.

The decision: Major correction is required

Best regards,

Luma Al Obaidy
