# Supplementary material for: A multilayered genetic structure analysis between inflammatory bowel disease and bone density/osteoporosis
Source: PLoS One. 2025 Nov 20;20(11):e0336775. doi: 10.1371/journal.pone.0336775 (PMC12633866; doi:10.1371/journal.pone.0336775)
Supplement: Attachment — Submitted filename: To the editor PONE-D- 25-31485 R2.pdf [file pone.0336775.s006.pdf]

University of Baghdad/
College of Science for Women
Biology department
Baghdad
10 /25 /2025

Dear Editors in PLOS ONE

I hope you are fine.

Thank you to give me the opportunity to review the manuscript, PONE-D-25-31485, entitled (A Multilayered Genetic Structure Analysis between Inflammatory Bowel Disease and Bone Density/Osteoporosis).

The study focuses on the wide genomic data analysis of the association between inflammatory bowel disease (IBD) and reduced bone mineral density (BMD) or osteoporosis.

The study analyzes a large cohort of the UK biobank patients with Inflammatory bowel disease.

The decision**: Acceptance** , as the authors explained the queries and added the requested notes.

Best regards,

Luma Al Obaidy
