# Supplementary material for: A multilayered genetic structure analysis between inflammatory bowel disease and bone density/osteoporosis
Source: PLoS One. 2025 Nov 20;20(11):e0336775. doi: 10.1371/journal.pone.0336775 (PMC12633866; doi:10.1371/journal.pone.0336775)
Supplement: Attachment — Submitted filename: Response to Reviewers.docx [file pone.0336775.s004.docx]

**RESPONSE LETTER**

Oct. 05, 2025

Dear Prof. Emmanuel O Adewuyi, BPharm,

Thank you for reviewing our manuscript (Manuscript ID: **PONE-D-25-31485**; Manuscript title: **A Multilayered Genetic Structure Analysis between Inflammatory Bowel Disease and Bone Density/Osteoporosis**) and for your decision of further consideration. Now we have revised and checked the manuscript carefully, according to the editor’s and reviewer’s comments.

We submit here the revised paper. The changes and explanations regarding the revisions of our paper are listed below this letter.

We have tried our best to address all the concerns raised by the editor and reviewer. We hope that, with these modifications and improvements based on the editor’s and reviewer’s comments, our manuscript would be acceptable for publication in your journal.

Thank you again!

With best wishes,

Mengting Qin

Department of Anorectal Surgery, Zhejiang Chinese Medical University Affiliated Jiaxing TCM Hospital, Jiaxing, China

Email: [qinmt0907@163.com](mailto:qinmt0907@163.com)

**Reply to Editor**

Thank you for submitting your manuscript to PLOS ONE. After careful consideration, we feel that it has merit but does not fully meet PLOS ONE’s publication criteria as it currently stands. Therefore, we invite you to submit a revised version of the manuscript that addresses the points raised during the review process.

**Response:**

Thank you for your feedback and for considering our manuscript.

We have carefully reviewed the reviewer's comments and appreciate their valuable feedback. We acknowledge the importance of enhancing the clarity and understanding of our work, and we have already implemented the suggested modifications to address these concerns (please see the point-by-point response below for details).

Thank you again!

**ACADEMIC EDITOR:**

#1:Reviewers have provided feedback. Authors need to pay close attention and respond to the comments, making corrections to their manuscript as necessary. As noted by some of the reviewers, there is a need to strengthen methodological rigour and provide more detail about the data utilised.

**Response:**

We sincerely thank the reviewer for the constructive feedback. In response to the comments, we have revised the manuscript to strengthen the methodological clarity and provided additional details regarding the data utilized. The specific changes are summarized below.

#2:Is there a reason the authors did not perform Mendelian randomisation to assess potential causal associations? Yes, I see a reference to previous studies, but could they have assessed this to confirm or contextualise their findings? It would probably add depth and context to their study.

**Response:**

We sincerely thank the academic editor for this valuable suggestion. We have taken it into account and included a Mendelian randomisation analysis in the revised manuscript (see sections 2.4, **lines 161-171**, and 3.3, **lines 264-276**). The results of this analysis are consistent with previous studies, and we believe that this addition has enhanced the depth and context of our findings. We greatly appreciate the editor’s contribution to improving the manuscript.

**Reply to Reviewer 1**

**In the methods:**

**#1:** The total number of participants in the study did not specify the number of males and females or the participants age distribution to identify the more affected sex and age.

**Response:**

We sincerely thank the reviewer for the valuable suggestion. We have now provided additional details regarding the cohort information in the revised manuscript, including Phenotypes, Phenotypic code, Cases/Controls, and Ancestry (see Table 1, **lines 695-697**). We acknowledge that the current study did not specify the number of males and females or the participants' age distribution. This is due to the lack of sex- and age-stratified data in our current dataset, which limited our ability to explore sex- and age-specific genetic susceptibility to IBD, BMD, and osteoporosis comorbidity. Therefore, we recognize that the absence of sex and age stratification analysis is a limitation of this study.

Additionally, it is important to note that most current GWAS studies have not conducted sex- and age-stratified analyses, which is not unique to our research. In this study, we made efforts to analyze the data from multiple levels (local and global) and angles (GNOVA, HDL, LAVA, cond/conjFDR). Our methodological results were consistent across these approaches, which in part compensates for the lack of sex- and age-stratified analysis.

Furthermore, in the limitations section, we mention that if more sex- and age-stratified data become available, we plan to incorporate sex-specific and age-stratified analyses in future studies to further investigate the role of sex and age in IBD, BMD, and osteoporosis comorbidity (please see **lines 412-415** of the revised manuscript).

***Lines 412-415***: The GWAS datasets used in this study were not stratified by sex and age, limiting our ability to explore sex- and age-specific genetic susceptibility to IBD, BMD, and osteoporosis comorbidity. Future analyses will address this limitation if such data become available.

**#2:** As the IBD (Crohn’s disease (CD) and ulcerative colitis (UC)) is mentioned in lines 32 and 33, it is expected the immune response genes are involved, but genes that govern the immune response, like HLA genes, or genes related to the absorption and metabolism of calcium, potassium, phosphorus, etc., and genes related to vitamin D, such as VDR, etc., are not analyzed in the study, which may have an important impact on bone density.

It will be a significant addition if further genes are included and their role in bone density is elucidated in a large number of populations and categorized according to their sex and age.

**Response:**

We sincerely thank the reviewer for the valuable suggestion. We understand that immune response genes (such as HLA genes), genes related to the absorption and metabolism of calcium, potassium, phosphorus, etc., and genes related to vitamin D metabolism (such as VDR) may play an important role in the relationship between IBD and bone density. In our current study, the focus was to establish the overall and local genetic associations between IBD and BMD/osteoporosis through GNOVA, HDL, and LAVA analyses. We also identified shared genetic loci between IBD and BMD/osteoporosis through CondFDR/ConjFDR analysis, including genes like ZBTB40 and RSPO3, which have been repeatedly validated. Therefore, in the discussion section (**lines 353-385**), we focused on the potential mechanisms of these genes in the context of IBD and BMD/osteoporosis. Based on current literature, ZBTB40 is indeed closely related to immune response, vitamin D metabolism, and calcium ion balance (1,2). RSPO3, on the other hand, is associated with bone density and immune processes (3,4).

Regarding the stratified analysis by sex and age, we have provided a detailed explanation in response to **comment #1**. We sincerely appreciate the reviewer’s guidance and valuable input.

**Reply to Reviewer 2**

**Abstract**

#1:Clarify “biologically significant” loci. Does this mean functional annotation, pathway relevance, or previous association?

**Response:**

We sincerely thank the reviewer for the valuable question. In this study, “biologically significant” loci refer to those identified based on functional annotation, pathway analysis, and prior associations with traits or diseases. Using conditional/conjunctional false discovery rate (cond/conjFDR) statistical methods, we identified several biologically significant shared genetic loci, which are defined by their functional annotation, relevance to known biological pathways, and prior associations with traits or diseases (5).

Through functional annotation, we found that these loci are enriched in the Wnt signaling pathway. Additionally, we referred to previous studies to confirm that genes such as ZBTB40 and RSPO3 are involved in important biological processes. ZBTB40 plays a critical role in the immune system, vitamin D metabolism, and mineral metabolism, while RSPO3 is closely associated with bone density regulation and immune function. These details are further discussed in the manuscript (**lines 353-406**).

#2:Add the total number of GWAS datasets or total sample size used for clarity.

**Response:**

We thank the reviewer for the suggestion. The total number of GWAS datasets and sample size has been added for clarity, as seen in **lines 15-16** of the revised manuscript.

***Lines 15-16***: Utilizing data from genome-wide association studies on 59,957 IBD, 40,266 CD, 45,975 UC, 31,492 BMD, and 399,054 osteoporosis,

**Introduction**

#1:Update references to include more recent global epidemiological data where possible.

**Response:**

We sincerely thank the reviewer for the suggestion. In response, we have updated the references with more recent global epidemiological data, as indicated in **lines 39-42 and 48-49** of the revised manuscript.

***Lines 39-42***: Recent 2025 studies show that IBD has entered a "high prevalence stage," with both incidence and prevalence at elevated levels. In Western countries, the prevalence has reached or is near 1%, and by 2030, over 1% of the population is expected to be affected, putting significant strain on healthcare systems (6).

***Lines 49-50***: Osteoporosis affected 41.5 million people globally in 2019, with a projected 263.2 million cases by 2034 (7).

#2:The paragraph beginning on line 51 is overly dense. Consider splitting into two, and briefly introduce each analytic tool in one sentence before elaborating.

**Response:**

We sincerely thank the reviewer for the helpful suggestion. In response, we have split the paragraph into three and briefly introduced each analytic tool in one sentence before elaborating. The revisions can be found in **lines 54-78** of the revised manuscript.

**Methods and Materials**

#1:Specify the exact sample size for each GWAS used and any data pre-processing or quality control performed (SNP filtering, ancestry exclusion) (Line 84–91).

**Response:**

We sincerely thank the reviewer for the helpful comment. In response, we have added the exact sample sizes for each GWAS dataset, including the number of cases, controls, and the total sample size (for BMD, as a continuous variable, we have provided the total sample size instead of case/control counts) in **lines 93-100**. We have also included Table 1 (**lines 695-697**), which provides details such as Phenotypic code, Cases/Controls, Ancestry, etc. Furthermore, we have added information on data pre-processing and quality control, including SNP filtering and ancestry exclusion, in **lines 101-108**.

#2:State the specific genomic resolution used in the LAVA (e.g., 1Mb blocks? predefined regions?). Also, clarify if any Bonferroni or FDR correction was applied in interpreting local correlations (Line 120–130).

**Response:**

We sincerely thank the reviewer for the insightful comments. In response, we clarify that the specific genomic resolution used in the LAVA analysis is indeed 1Mb blocks (8), as indicated in the revised manuscript (**lines 146-147**). Additionally, we have included the details regarding the statistical corrections applied to all methods used in this study. These corrections, including Bonferroni and FDR adjustments, are described in **lines 109-115** of the manuscript. We hope this addresses your concerns, and we appreciate your helpful feedback.

***Lines 109-115***: To rigorously control the false-positive rate in the analysis, this study applied specific statistical correction strategies for each method. For the genome-wide genetic correlation analyses conducted using GNOVA, HDL, and MR, we applied the Bonferroni correction. In the case of LAVA analysis, p-values were adjusted using the Benjamini-Hochberg (BH) method to effectively control the false discovery rate (FDR) and identify significant associations. In the condFDR/conjFDR analyses, a significance threshold of conjFDR < 0.05 was adopted, consistent with the practices commonly used in previous studies (9–12).

#3:Clarify whether the FUMA gene annotations were only positional or included eQTL, chromatin interactions, etc.

**Response:**

We sincerely thank the reviewer for the valuable comment. The FUMA gene annotations were not limited to positional information; they also included gene expression data, chromatin interactions, and other relevant information. In addition, we have provided further details on how the results obtained through conjFDR analysis were annotated using FUMA, which can be found in **lines 204-219** of the revised manuscript. We hope this clarification addresses your concerns, and we appreciate your helpful feedback.

**Results**

#1:Enrichment Results (lines 216–231): Clarify how significance was defined in GO and KEGG enrichment (e.g., FDR < 0.05?).

**Response:**

We sincerely thank the reviewer for the valuable comment. In response to your suggestion, we have added the methodological details for the GO and KEGG enrichment analysis in the revised manuscript, which can be found in Section 2.7 (**lines 220-226**). We clarified that significance in the GO and KEGG enrichment analysis was defined using an FDR threshold of < 0.1. We hope this addresses your concern, and we appreciate your helpful feedback.

#2:Were any of the identified shared loci previously known in either disease, or are they novel findings?

**Response:**

We sincerely thank the reviewer for the valuable question. In our study, some of the identified shared genetic loci have been previously associated with IBD. For example, genes like ZBTB40 and RSPO3 have been shown to be involved in immune response and vitamin D metabolism (1–4). However, we also identified several novel genetic loci, which can be found in Supplementary Table S7-12. In the table, if the “novel gene” column indicates “YES,” it refers to newly identified genes that have not been widely reported in IBD or other related diseases. If the column indicates “NO,” the gene is known. We believe these new findings offer promising directions for further exploration of the genetic mechanisms underlying IBD.

**References**
#1:Ensure all URLs (e.g., for software tools like FUMA, Sangerbox) are active and appropriately cited. (Line 350-351) “All GWAS data and statistical software utilized in this study were publicly available (accessible via the following URLs)…”

**Response:**

We sincerely thank the reviewer for the valuable suggestion. We have now included the URLs for accessing the GWAS data and statistical software used in this study (**lines 433-442**), and have ensured that they are appropriately cited in the manuscript. We hope this addresses your concern, and we appreciate your helpful feedback.

**Reply to Reviewer 3**

The manuscript by Qin et al. has explored the association between inflammatory bowel disease (IBD) and bone mineral density (BMD) and osteoporosis with extensive statistical tests, and concluded that a notable genetic correlation exists between IBD and BMD/osteoporosis.

The introduction was well written with clear explanation of different statistical methods for GWAS testing and Rg analysis. And the discussion went to great lengths to review the genes/pathways and their potential roles in the development of IBD/BMD/osteoporosis.

However, there are a few questions regarding the design and interpretation of the work, hope the authors could address before considered for publication:

**Response:**

We sincerely thank the reviewer for their positive feedback and recognition of the clarity in the introduction and thoroughness of the discussion. We also appreciate the constructive points raised regarding the study’s design and interpretation. Below, we provide responses to address the concerns raised and hope the revisions will meet your expectations for publication.

#1:Since the study was solely based on the statistical testing and computational simulation of the GWAS data, could the authors provide more details and characterization of the data? I.e. the sample size, age group, stage of IBD/osteoporosis etc., as well as the features that were selected for statistical testing, which would affect the interpretation of correlation scores.

**Response:**

We sincerely thank the reviewer for the valuable comment. In our study, the GWAS data used covered 59,957 IBD patients, 40,266 Crohn's disease (CD) patients, 45,975 ulcerative colitis (UC) patients, 31,492 bone mineral density (BMD) samples, and 399,054 osteoporosis patients (**lines 93-99**). The specific characteristics of the samples and related information can be found in Table 1 of the manuscript (**lines 695-697**).

Regarding age groups, disease stages of IBD/osteoporosis, and other information, the raw data used in this study were not stratified by sex, age, or disease stage, and therefore, we were unable to analyze these aspects in detail. We have added this limitation to the manuscript in the limitations section (**lines 412-415**). The data used in this study are primarily from European populations, which also limits the generalizability of our findings to other ethnic groups. All data were obtained from publicly available GWAS databases and underwent strict quality control to ensure accuracy and reliability. The features selected for statistical testing include genetic variants, SNP frequencies, etc. (**lines 101-108**), which are crucial for the interpretation of the correlation scores.

We hope these additional details address the reviewer’s concerns, and we appreciate your valuable guidance and suggestions.

#2:Have the authors taken into account possible confounding factors that could cause spurious correlations? I.e. both osteoporosis and IBD (especially CD) has age-related manifestation, which might require additional testing or randomization to rule out the cofounding effect.

**Response:**

We sincerely thank the reviewer for raising this important issue. We understand that age is a critical factor influencing both IBD and osteoporosis, especially since both IBD (particularly Crohn's disease) and osteoporosis are age-related, which may influence the correlation results. In our study, due to the lack of stratification by sex, age, or disease stage in the GWAS data, age and other factors could indeed affect the interpretation of the correlation scores. We have addressed this limitation in the revised manuscript (**lines 412-415**).

It is worth noting that we used multiple statistical methods (GNOVA, HDL, LAVA, and conjfdr) for the GWAS correlation analysis of IBD, BMD, and osteoporosis, which, compared to clinical observational studies, helps to limit the influence of confounding factors. This is one of the advantages of GWAS (13). Furthermore, we have added a Mendelian randomisation analysis to the revised manuscript, which is another statistical method that can help avoid confounding factors (14). The results of this analysis are highly consistent with previous studies (15–17).

In summary, we believe the results of our study regarding the genetic correlation between IBD and osteoporosis are reliable and trustworthy. Thank you once again for your valuable input.

#3:To further validate the findings, could the author include negative controls into the testings (i.e. using unrelated/negative samples, or check against unrelated symptoms)?

**Response:**

We sincerely thank the reviewer for the thoughtful suggestion. In our study, we employed a variety of robust statistical methods, including GNOVA and HDL to explore the overall genetic correlation between IBD, BMD, and osteoporosis, LAVA to investigate their local genetic correlations, Mendelian randomization to examine causal relationships, and CondFDR/ConjFDR analyses to assess genetic overlap and identify shared loci. Furthermore, we approached the study from both the BMD and osteoporosis perspectives, and the results demonstrated a high degree of consistency across different analyses, reinforcing the reliability of our findings.

Given that GWAS-based approaches, as used in our study, help to mitigate confounding effects more effectively than observational studies (13), we believe the current findings are sufficiently validated. Therefore, we respectfully believe that the inclusion of additional negative controls is not necessary for further validation of the results. We hope this explanation addresses the reviewer’s concern, and we are grateful for your valuable input and constructive feedback.

**Reply to Reviewer 4**

In this paper, the authors utilized data from GWAS on IBD, its subtypes, BMD, and osteoporosis to conduct comprehensive analyses with focuses on genetic correlations and shared genetic loci. In addition, local genetic patterns were examined using local variant association analysis. As a result, a notable genetic correlation was detected between IBD, its subtypes, and BMD/osteoporosis. Overall, the paper is well-written. Below are my comments on the paper.

**Response:**

We sincerely thank the reviewer for the positive evaluation of our manuscript. We greatly appreciate your recognition of the clarity and quality of our work. Your thoughtful feedback and comments are highly valued, and we are grateful for the time you have spent reviewing our paper. Below, we provide point-by-point responses to address the concerns raised. We hope our revisions meet your expectations and further strengthen the manuscript.

#1:• In Section 3.2, the authors mentioned LAVA identified specific chromosomal segments associated with IBD and BMD. I would recommend the authors to clearly indicate how the chromosomal segments are determined either in section 2.3 or in section 3.2.

**Response:**

We sincerely thank the reviewer for the valuable comment. In response, we have provided additional details on how the chromosomal segments are determined in Section 2.3, which can be found in **lines 149-152** of the revised manuscript. We hope this clarification addresses your concern, and we appreciate your constructive feedback.

***Lines 149-152***: To identify chromosomal segments associated with IBD and BMD, LAVA selects specific genomic regions based on predefined 1Mb blocks. These blocks are defined by genomic coordinates, and their selection is optimized to minimize LD between adjacent blocks, ensuring independent genetic signals are captured.

#2:• The resolutions of the figures in the manuscript needs improvement. For instance, it is hardly to see any numbers and labels on the horizontal and vertical axes in Figure 2.

**Response:**

We sincerely thank the reviewer for the valuable feedback. In response, we have reprocessed all the figures in the manuscript to improve their resolution, making them clearer and ensuring that the numbers and labels on the horizontal and vertical axes are easily visible. We hope these improvements address your concern, and we appreciate your constructive suggestions.

**Reply to Reviewer 5**

Reviewer #5: The authors conducted a series of genetic analyses to investigate the shared genetic contribution to IBD and bone mineral density (BMD)/osteoporosis. Through integration of their findings, they identified four genes that may be regulated by variants associated with both IBD and BMD/osteoporosis. The approach is interesting and potentially valuable for advancing our understanding of the shared genetic predisposition underlying these complex phenotypes.
However, the manuscript lacks in-depth interpretation and integration of the results obtained from the different analytical strategies. The use of global Rg, local Rg, and conjFDR is described, but the interplay between these methods—and whether their results corroborate one another—is not sufficiently explored. From this reviewer’s perspective, the results are primarily reported in a descriptive manner, with limited synthesis or biological insight. In addition, the manuscript suffers from insufficient methodological transparency, which limits reproducibility. For these reasons, the analyses require deeper investigation and clearer integration before the work can be considered for publication.

**Response:**

We sincerely thank the reviewer for the constructive feedback and for recognizing the potential value of our approach in advancing the understanding of shared genetic predisposition between IBD and BMD/osteoporosis. We appreciate the reviewer’s insightful comments regarding the need for deeper interpretation and integration of the results. Below, we provide point-by-point responses to address the concerns raised. We hope these revisions address the reviewer’s concerns and provide clearer integration of the results. We greatly appreciate your valuable suggestions and believe the revised manuscript is now stronger as a result.

1. The Methods section requires greater detail to ensure reproducibility:

a. It is not clear which LD matrix was used for the HDL and LAVA tools. Please specify, as these methods are sensitive to the structure of the reference LD matrix. Furthermore, was any filtering applied to the reference genetic data to ensure homogeneous European ancestry before calculating the LD matrix?

**Response:**

We sincerely thank the reviewer for the insightful comments. In response, we clarify that the LD matrix used for the HDL tool is based on data from 336,000 genetically unrelated British individuals from the UK Biobank, while the LD matrix used for the LAVA tool is based on the European population from the 1000 Genomes Project (Phase 3). We have added this information to the revised manuscript, which can be found in **lines 131-132** and **152-154**. Additionally, the GWAS data involved in this study underwent quality control and preprocessing to ensure homogeneous European ancestry before calculating the LD matrix, as detailed in **lines 101-108** of the revised manuscript. We hope this addresses your concerns, and we appreciate your constructive feedback.

b. For the Local Analysis of Variant Association, the manuscript vaguely refers to “user-defined genomic regions.” While the supplementary materials (Tables S1–S6) indicate that the analysis was performed on a set of predefined, non-overlapping genomic blocks with specific coordinates, the Methods section should explicitly describe how these regions were defined.

**Response:**

We sincerely thank the reviewer for the valuable feedback. In response, we have clarified in the Methods section how the user-defined genomic regions were determined. Specifically, the analysis was conducted on predefined, non-overlapping genomic blocks of 1Mb, which were selected based on genomic coordinates. These blocks were optimized to minimize linkage disequilibrium (LD) between adjacent blocks, ensuring that independent genetic signals were captured. The details of how these regions were defined and selected are now explicitly described in the revised manuscript (**lines 149-152**).

***Lines 149-152***: To identify chromosomal segments associated with IBD and BMD, LAVA selects specific genomic regions based on predefined 1Mb blocks. These blocks are defined by genomic coordinates, and their selection is optimized to minimize LD between adjacent blocks, ensuring independent genetic signals are captured.

c. The procedure for enrichment analysis is not described in the Methods. Some information appears in the Results (lines 216–219), but this should be moved to the Methods and expanded.

**Response:**

We sincerely thank the reviewer for the valuable comment. In response, we have added the procedure for enrichment analysis to the Methods section, specifically in Section 2.7 (**lines 220-226**). We have also expanded the description to provide more clarity on the process. We hope this revision addresses the reviewer’s concern, and we appreciate your constructive feedback.

d. The parameters used in FUMA for the SNP2gene module are not reported. Were specific variant or gene annotations applied in this step?

**Response:**

We sincerely thank the reviewer for the insightful comment. In response, we have added detailed information about the FUMA annotation process and the parameters used in the revised manuscript, which can be found in **lines 204-219**. Indeed, specific variant and gene annotations were applied in this step. We hope this clarification addresses the reviewer’s concern, and we appreciate your constructive feedback.

2. In the local Rg and ConjFDR analyses, the results obtained for the IBD subtypes (UC and CD) appear to largely recapitulate those observed when considering IBD as the overall phenotype. The authors should further discuss which loci show genetic correlation only at the subtype level versus those consistent across the broader IBD phenotype, and clarify which loci specifically overlap with the IBD-wide analysis. In addition, the overlap of genetic correlation results between BMD and osteoporosis should be addressed.

**Response:**

We sincerely thank the reviewer for the valuable comments. We would like to clarify that the primary focus of our study was to investigate the genetic associations between IBD and both BMD and osteoporosis, using these two traits as complementary perspectives. Since most current GWAS studies include IBD subtypes (CD and UC), we also considered these subtypes to ensure the comprehensiveness of our study. Our results show that, overall, the genetic associations between IBD, CD, UC, BMD, and osteoporosis are largely consistent. However, there are some subtle differences, such as the identification of RSPO3 in CD and ZBTB40 in UC. Given the focus of our study, we have emphasized the genetic associations at the IBD-wide level in our discussion.

Regarding sample overlap, we agree that it is a critical issue in large-scale cross-trait genetic studies, as it may lead to inflated false positives and biased evaluations of causal relationships and pleiotropy. We carefully considered this concern at the outset of the study. To minimize potential overlap, we selected BMD data from the IEU dataset and osteoporosis data from the FinnGen database. While we acknowledge that complete elimination of overlap cannot be guaranteed, we took steps to minimize it by choosing GWAS summary statistics from different sources. We assessed sample overlap using trait-wise correlation scores based on z-scores, assuming a mixture of null and non-null Gaussian distributions, with a threshold set at 0.05 (18). Our analysis showed an overlap score of 0.033 between BMD and osteoporosis, indicating that the overlap was within an acceptable range and unlikely to introduce spurious associations.

We hope these clarifications address the reviewer’s concerns. Thank you again for the thoughtful feedback.

3. Concordance between methods

Do the ConjFDR findings corroborate those of the local Rg analysis? Specifically, do the loci identified in local Rg include the variants highlighted by ConjFDR?

**Response:**

We sincerely thank the reviewer for the thoughtful question. We would like to clarify that the statistical principles behind the LAVA and CondFDR/ConjFDR analyses are distinct. LAVA identifies specific genomic regions with local genetic correlation between traits (8), while CondFDR and ConjFDR assess genetic overlap and identify shared genetic loci (19).

Our findings show that IBD and BMD/osteoporosis exhibit local genetic correlation, and the Q-Q plots suggest genetic overlap between the two traits, indicating some level of consistency between the methods. Notably, the ZBTB40 locus, identified in our analysis, is located on chromosome 1, and we found local genetic correlation between IBD and BMD on chromosome 1 as well. Similarly, RSPO3 is located on chromosome 6, where local genetic correlation between IBD and osteoporosis is also observed. These results suggest that the loci identified by local Rg analysis align with the findings from the ConjFDR analysis.

We have added a summary in the revised manuscript to highlight the consistency between the ConjFDR and LAVA results, which can be found in **lines 349-353** of the revised manuscript. Thank you again for your insightful suggestion.

***Lines 349-353***: The conjFDR method successfully identifies multiple significant genetic variation loci distributed across different chromosomal regions. A comprehensive analysis of the local genetic relationship between these two diseases suggests that certain chromosomal segments may play a crucial role in their shared pathogenic mechanisms, potentially regulated by functional genes located within these segments, such as ZBTB40 and RSPO3.

4. What did the FUMA analysis reveal? Were the identified variants located in regulatory regions, coding regions, or both?

**Response:**

We sincerely thank the reviewer for this insightful question. The FUMA analysis using the SNP2Gene module revealed that the identified variants were located in both regulatory and coding regions. We selected independent significant SNPs with condFDR < 0.05 and LD r² ≤ 0.6, excluding the MHC region to avoid interference (**lines 204-219**). The results also integrated gene expression and chromatin interaction data, providing a comprehensive understanding of the functional roles of the candidate genes. Thank you again for your thoughtful feedback.

5. The results of the enrichment analysis should be presented more clearly, ideally in supplementary tables. For example, which genes identified through ConjFDR and FUMA analyses are included within the enriched pathways?

**Response:**

We sincerely thank the reviewer for the valuable suggestion. In response, we have added detailed results of the enrichment analysis in the supplementary materials. These can be found in Supplementary Table 10-11 and Supplementary Table 15-16, which include the genes identified through the ConjFDR and FUMA analyses that are associated with the enriched pathways. We believe this addition enhances the clarity of our findings. Thank you again for your constructive feedback.

6. Why did the authors restrict their focus to variants and genes common to both BMD and osteoporosis? Were these shared genes enriched in specific pathways? How do the other identified genes (those not shared between BMD and osteoporosis) behave—do they also map to relevant pathways?

**Response:**

We sincerely thank the reviewer for the thoughtful comments. As mentioned in section “2.” of the manuscript, the primary focus of our study was to investigate the genetic associations between IBD and both BMD and osteoporosis, using these two complementary traits to identify shared genetic variants. We performed enrichment analysis on all shared loci (**lines 290-295, 302-304**), and in the discussion section, we extensively explored the role of the **Wnt signaling pathway** in bone metabolism regulation and gut homeostasis (**lines 386-406**). We hope this clarifies the reviewer’s concerns. Thank you again for your constructive feedback.

Minor comments:

1. In Figure 1, the workflow gives the misleading impression that the output of each analysis serves as the input for the subsequent one. I recommend revising the figure to clearly indicate that the input for all three analyses is the GWAS summary statistics.

**Response:**

We thank the reviewer for the suggestion. In response, we have revised Figure 1 to clearly show that the input for all three analyses is the GWAS summary statistics, ensuring better clarity of the workflow. Thank you again for your helpful feedback.

**Reference：**

1.	Chen L, Davey Smith G, Evans DM, Cox A, Lawlor DA, Donovan J, Yuan W, Day INM, Martin RM, Lane A, et al. Genetic variants in the vitamin d receptor are associated with advanced prostate cancer at diagnosis: findings from the prostate testing for cancer and treatment study and a systematic review. *Cancer Epidemiol Biomarkers Prev* (2009) 18:2874–2881. doi: 10.1158/1055-9965.EPI-09-0544

2.	Rioux JD, Xavier RJ, Taylor KD, Silverberg MS, Goyette P, Huett A, Green T, Kuballa P, Barmada MM, Datta LW, et al. Genome-wide association study identifies new susceptibility loci for Crohn disease and implicates autophagy in disease pathogenesis. *Nat Genet* (2007) 39:596–604. doi: 10.1038/ng2032

3.	Nagasawa T. CXC chemokine ligand 12 (CXCL12) and its receptor CXCR4. *J Mol Med (Berl)* (2014) 92:433–439. doi: 10.1007/s00109-014-1123-8

4.	Estrada K, Styrkarsdottir U, Evangelou E, Hsu Y-H, Duncan EL, Ntzani EE, Oei L, Albagha OME, Amin N, Kemp JP, et al. Genome-wide meta-analysis identifies 56 bone mineral density loci and reveals 14 loci associated with risk of fracture. *Nat Genet* (2012) 44:491–501. doi: 10.1038/ng.2249

5.	Wang S, Dan Y-L, Yang Y, Tian Y. The shared genetic etiology of antisocial behavior and psychiatric disorders: Insights from pleiotropy and causality analysis. *J Affect Disord* (2024) 365:534–541. doi: 10.1016/j.jad.2024.08.149

6.	Kaplan GG. The global burden of inflammatory bowel disease: From 2025 to 2045. *Nat Rev, Gastroenterol Hepatol* (2025) 22:708–720. doi: 10.1038/s41575-025-01097-1

7.	Sex specific global burden of osteoporosis in 204 countries and territories, from 1990 to 2030: An age-period-cohort modeling study. *J Nutr, Health Aging* (2023) 27:767–774. doi: 10.1007/s12603-023-1971-4

8.	Werme J, van der Sluis S, Posthuma D, de Leeuw CA. An integrated framework for local genetic correlation analysis. *Nat Genet* (2022) 54:274–282. doi: 10.1038/s41588-022-01017-y

9.	Huang Z, Yuan W. Exploring genetic structures and shared sites between alcohol, cheese intake, and inflammatory bowel disease. *Front Nutr* (2025) 12:1468457. doi: 10.3389/fnut.2025.1468457

10.	Lu W, He X, Peng H, Lei P, Liu J, Ding Y, Yan B, Ma X, Yang J. Exploring the shared genetic architecture between testosterone traits and major depressive disorder. *BMC Psychiatry* (2025) 25:651. doi: 10.1186/s12888-025-07096-5

11.	Karadag N, Hagen E, Shadrin AA, van der Meer D, O’Connell KS, Rahman Z, Kutrolli G, Parker N, Bahrami S, Fominykh V, et al. Dissecting the shared genetic architecture of common epilepsies with cortical brain morphology. *Neurol, Genet* (2024) 10:e200143. doi: 10.1212/NXG.0000000000200143

12.	Ou Y-N, Wu B-S, Ge Y-J, Zhang Y, Jiang Y-C, Kuo K, Yang L, Tan L, Feng J-F, Cheng W, et al. The genetic architecture of human amygdala volumes and their overlap with common brain disorders. *Transl Psychiatry* (2023) 13:90. doi: 10.1038/s41398-023-02387-5

13.	Uffelmann E, Huang QQ, Munung NS, de Vries J, Okada Y, Martin AR, Martin HC, Lappalainen T, Posthuma D. Genome-wide association studies. *Nat Rev Methods Primers* (2021) 1:59. doi: 10.1038/s43586-021-00056-9

14.	Emdin CA, Khera AV, Kathiresan S. Mendelian Randomization. *JAMA* (2017) 318:1925–1926. doi: 10.1001/jama.2017.17219

15.	Kang J, Wu X, Li Y, Zhao S, Wang S, Yu D. Association between inflammatory bowel disease and osteoporosis in European and East Asian populations: exploring causality, mediation by nutritional status, and shared genetic architecture. *Front Immunol* (2024) 15:1425610. doi: 10.3389/fimmu.2024.1425610

16.	Dai Z, Xu W, Ding R, Peng X, Shen X, Song J, Du P, Wang Z, Liu Y. Two-sample Mendelian randomization analysis evaluates causal associations between inflammatory bowel disease and osteoporosis. *Front Public Health* (2023) 11: doi: 10.3389/fpubh.2023.1151837

17.	Xu D, Chen Y, Gao X, Xie W, Wang Y, Shen J, Yang G, Xie B. The genetically predicted causal relationship of inflammatory bowel disease with bone mineral density and osteoporosis: evidence from two-sample Mendelian randomization. *Front Immunol* (2023) 14:1148107. doi: 10.3389/fimmu.2023.1148107

18.	Smeland OB, Frei O, Shadrin A, O’Connell K, Fan C-C, Bahrami S, Holland D, Djurovic S, Thompson WK, Dale AM, et al. Discovery of shared genomic loci using the conditional false discovery rate approach. *Hum Genet* (2020) 139:85–94. doi: 10.1007/s00439-019-02060-2

19.	Smeland OB, Frei O, Shadrin A, O’Connell K, Fan C-C, Bahrami S, Holland D, Djurovic S, Thompson WK, Dale AM, et al. Discovery of shared genomic loci using the conditional false discovery rate approach. *Hum Genet* (2020) 139:85–94. doi: 10.1007/s00439-019-02060-2
